# Updates on clinical studies of selenium supplementation in radiotherapy

**DOI:** 10.1186/1748-717X-9-125

**Published:** 2014-05-29

**Authors:** Irma M Puspitasari, Rizky Abdulah, Chiho Yamazaki, Satomi Kameo, Takashi Nakano, Hiroshi Koyama

**Affiliations:** 1 Department of Public Health, Gunma University Graduate School of Medicine, 3-39-22 Showa Machi, Maebashi 371-8511, Japan; 2 Department of Pharmacology and Clinical Pharmacy, Faculty of Pharmacy, Universitas Padjadjaran, Bandung, Indonesia; 3 Department of Radiation Oncology, Gunma University Graduate School of Medicine, Gunma, Japan

**Keywords:** Selenium, Supplementation, Clinical studies, Radiotherapy

## Abstract

To establish guidelines for the selenium supplementation in radiotherapy we assessed the benefits and risks of selenium supplementation in radiotherapy. Clinical studies on the use of selenium in radiotherapy were searched in the PubMed electronic database in January 2013. Sixteen clinical studies were identified among the 167 articles selected in the initial search. Ten articles were observational studies, and the other 6 articles reported studies on the effects of selenium supplementation in patients with cancer who underwent radiotherapy. The studies were conducted worldwide including European, American and Asian countries between 1987 and 2012. Plasma, serum or whole blood selenium levels were common parameters used to assess the effects of radiotherapy and the selenium supplementation status. Selenium supplementation improved the general conditions of the patients, improved their quality of life and reduced the side effects of radiotherapy. At the dose of selenium used in these studies (200–500 μg/day), selenium supplementation did not reduce the effectiveness of radiotherapy, and no toxicities were reported. Selenium supplementation may offer specific benefits for several types of cancer patients who undergo radiotherapy. Because high-dose selenium and long-term supplementation may be unsafe due to selenium toxicity, more evidence-based information and additional research are needed to ensure the therapeutic benefits of selenium supplementation.

## Introduction

Radiotherapy is one of the most common and effective treatments for cancer [1]. Radiation damages cancer cells by direct ionization of DNA and by indirect effects caused by reactive oxygen species (ROS) [2]. Ionizing radiation consists of electromagnetic radiations, including X-rays and gamma rays, and particulate radiation such as electrons, protons and neutrons [2]. Exposure to ionizing radiation produces ROS in the tissue environment; including hydroxyl radicals (the most damaging), superoxide anion radicals and other oxidants such as hydrogen peroxide [2]. Although radiotherapy is effective in killing cancer cells, ROS produced in radiotherapy may threaten the integrity and survival of the surrounding normal cells and may cause late side effects of radiotherapy [1-3]. The administration of radioprotective agents, which are supposed to scavenge radiation-induced radicals and reduce the effects of radiation at an early stage, has been suggested as one approach for prophylaxis of radiation effects in normal tissues [4,5].

Selenium, a trace element, is an essential nutrient of fundamental importance in human biology [6] and as a preventive approach to ROS detoxification, which activates and stimulates the endogenous system [4,7,8]. Some of the most fundamental cellular processes, such as DNA synthesis, depend on the presence of selenium within the catalytic site of thioredoxin reductases (TrxR) [9,10]. A moderate deficiency of selenium has been linked to many conditions, such as an increased risk of cancer, infections and male infertility; a decrease in immune and thyroid function; and several neurological conditions [6,9]. A review paper reported that in prospective studies published in the 1980s and early 1990s involving 8,000 to 11,000 individuals, low selenium status was associated with significantly increased risks of cancer incidence and mortality [6].

A number of mechanisms have been suggested to explain the anti-cancer effects of selenium [11]. Selenium in selenoproteins can reduce oxidative damage and can limit DNA damage, both of which are linked to cancer risk [11]. Other cellular processes and molecular pathways that may be involved in the anti-cancer effects of selenium are the induction of phase II conjugating enzymes that detoxify carcinogens, enhancement of the immune response, an increase in tumor-suppressor protein p53, inactivation of protein kinase C (PKC), alterations in DNA methylation, blockage of the cell cycle to allow DNA repair, induction of apoptosis in cancer cells and inhibition of angiogenesis [11]. In survey studies, selenium has been reported as a complementary alternative medicine (CAM) used in lung and prostate cancer patients undergoing radiotherapy [12,13].

However, there are no guidelines on selenium supplementation in radiotherapy which should consist of inclusion and exclusion criteria for selenium supplementation, applicable cancer types, dose of supplementation, chemical form of selenium, duration of supplementation and the possible side effects of supplementation in radiotherapy. First, however, the benefits and risks of selenium use in radiotherapy should be clarified, as such information is still insufficient. Tabassum et al. [14] summarized the protective effect of selenium against prostate cancer and Fritz et al. [15] reviewed the relationship between selenium and lung cancer, suggesting positive effects of selenium in radiotherapy. Dennert and Horneber reviewed two clinical trials published in 2006 as a Cochrane database systematic review, and the review was revised in 2011 with the addition of one trial [16]. The subjects in the Cochrane database systematic reviews were limited to those included in randomized controlled trials; therefore, only 3 studies were reviewed, yielding no clear evidence that selenium supplements improve the side effects of cancer therapy [16]. In this paper, we summarized the clinical studies on selenium and radiotherapy to provide evidence-based information on the benefits and risks of selenium supplementation to aid in the establishment of guidelines for selenium supplementation in radiotherapy.

## Methodology

**Figure 1:**
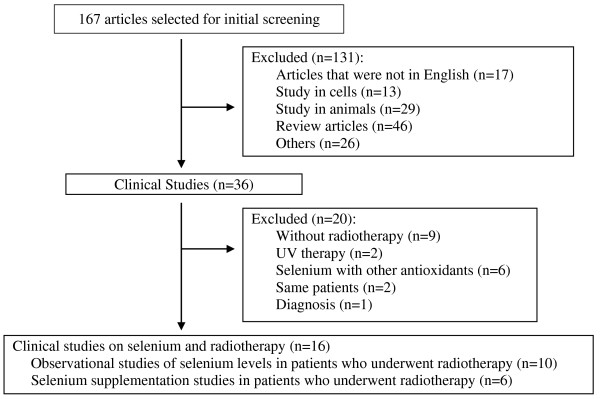
Flowchart of the literature search.

The flowchart of our literature search is shown in Figure 1. Briefly, a PubMed electronic database search using medical subject headings (MeSH) terms and the keywords “selenium”, “radiation” and “therapy” in January 2013 yielded 167 articles, 16 of which were clinical studies on selenium and radiotherapy. The detailed keyword search was as follows: “selenium” [MeSH Terms] OR “selenium” [All Fields]) AND (“radiotherapy” [Subheading] OR “radiotherapy” [All Fields] OR (“radiation” [All Fields] AND “therapy” [All Fields]) OR “radiation therapy” [All Fields] OR “radiotherapy” [MeSH Terms] OR (“radiation” [All Fields] AND “therapy” [All Fields]) OR “radiation therapy” [All Fields]).

### Clinical studies on selenium and radiotherapy

Table 1 lists the 16 articles on selenium and radiotherapy in clinical studies. The studies were conducted worldwide, including European, American and Asian countries, between 1987 and 2012. A total of 1303 patients undergoing radiotherapy participated in the studies. Of the 16 articles, 10 articles were observational studies investigating patients’ selenium levels before, during and/or after radiotherapy, while the other 6 articles investigated the effects of selenium supplementation studies on cancer patients who underwent radiotherapy. The cancer types investigated were upper gastrointestinal, breast, lung, larynx, head and neck, non-Hodgkin lymphoma, brain, oral, prostate and gynecological cancers.

**Table 1:** Clinical studies on selenium and radiotherapy between 1987 and 2012

**Study no.**	**Reference**	**Site of research**	**Number of patients**	**Study design**	**Type of cancer/disease**	**Radiotherapy delivery method**
1	Pothier et al. [17]	USA	n = 71	Observation of Se levels	Upper gastrointestinal cancer	Not mentioned
2	Antila et al. [18]	Finland	n = 24	Observation of Se levels	Breast cancer	Energy photons followed by 6–12 MeV electrons
3	Piccinini et al. [19]	Italy	n = 66	Observation of Se levels	Breast cancer (n = 38), lung cancer (n = 28)	Not mentioned
4	Rostkowska-Nadolska et al. [20]	Poland	n = 78	Observation of Se levels	carcinoma of the larynx	X-Ray therapy
5	Yadav et al. [21]	India	n = 30	Observation of Se levels	Head and neck cancer	Not mentioned
6	Last et al. [22]	UK	n = 100	Observation of Se levels	Non-Hodgkin’s lymphoma	Not mentioned
7	Fraunholz et al. [23]	Germany	n = 224	Observation of Se levels	Breast cancer (n = 94); cervical csncer (n = 25); head and neck cancer (n = 23); lung cancer (n = 19); prostate (n = 13); other (n = 50)	6-MV and/or 25-MV photons generated by a linear accelerator
8	Franca et al. [24]	Brazil	n = 209	Observation of Se levels	Breast cancer	Not mentioned
9	Zeng YC et al. [25]	China	n = 95	Observation of Se levels	Non-small cell lung cancer with brain metastases	6MV external beam radiotherapy
10	Eroglu C et al. [26]	Turkey	n = 47	Observation of Se levels	Head and neck cancer	Not mentioned
11	Pakdaman, [27]	Germany	n = 32	Se supplementation	Brain tumor	Not mentioned
12	Kiremidjian-Schumacher et al. [28]	USA	n = 33 supplemented (n = 17) placebo (n = 16)	Se supplementation	Head and neck cancer	Not mentioned
13	Micke et al. [29]	Germany	n = 48	Se supplementation	Secondary lymphedema	a linear accelerator with 6-MeV photons or a ^60^Co treatment unit
14	Elango et al. [30]	India	n = 126	Se supplementation	Oral cancer	a tele-cobalt beam (Theratron-780-^60^Co; phoenix-^60^Co; Gammatron-^60^Co)
15	Muecke et al. [31]	Germany	n = 81 supplemented (n = 39) control group (n = 42)	Se supplementation	Cervical cancer (n = 11); uterine cancer (n = 70)	6- to 18-MV linear accelerator
16	Buntzel et al. [32]	Germany	n = 39	Se supplementation	Head and neck cancer	Not mentioned
			Total = 1303			

Among the 16 articles, only 7 articles mentioned the types of radiotherapy delivered to the patients, and in most cases, electromagnetic radiations was used.

#### Observational studies

Table 2 presents the observational studies investigating the selenium levels in patients who underwent radiotherapy without selenium supplementation. In addition to the 10 observational studies, 2 studies assessed selenium levels in patients who did not received selenium supplementation, who served as a placebo group and control group in the selenium supplementation studies. Thus, a total of 12 studies measured the selenium levels in patients who underwent radiotherapy without selenium supplementation.

**Table 2:** Observational studies investigating the selenium levels in patients who underwent radiotherapy without selenium supplementation

**Study no.**	**Reference**	**Sample**	**Measurement method**	**Mean selenium levels (μg/l)**					
				**Before radiotherapy**	**During radiotherapy**		**End of radiotherapy**	**After completion of radiotherapy**	
1	Pothier et al. [17]	Plasma	AAS	61.8 (at stable stage)	1 week (n = 5)	34.3 **↓**	-	-	-
2	Antila et al. [18]	Serum	AAS	130.7*	Middle of therapy	124.4*	128.3*	2 weeks	126.7*
								2 months	121.2*
3	Piccinini et al. [19]	Plasma	Fluorometric method	Breast cancer: 71.81	-	-	-	-	-
				Lung cancer:69.59					
4	Rostkowska-Nadolska et al. [20]	Serum	AAS	253.8**	-	-	-	6 weeks	267.5**
5	Yadav et al. [21]	Serum	AAS	62.7	-	-	61.0	1 year	Cured:91.5
									Residual disease: 61.8
6	Last et al. [22]	Serum	ICP-MS	72.44*	-	-	-	-	-
7	Fraunholz et al. [23]	Whole blood	AAS	75.64	-	-	75.88	6 weeks	81.28
8	Franca et al. [24]	Plasma	AAS	≤60 years:101.8	-	-	≤60 years: 58.1↓	-	-
				>60 years:65.2			>60 years: 33.7↓		
9	Zeng YC et al. [25]	Whole blood	AAS	90.4	-	-	56.3↓	-	-’
10	Eroglu C et al. [26]	Serum	ICP-MS	58.09	-	-	56.34	-	-
12	Kiremidjian-Schumacher et al. [28]	Plasma (placebo group)	Graphite-furnace AAS	94.38	-	-	8th weeks: 91.8	8 weeks	89.92
15	Muecke et al. [31]	Whole blood (control group)	AAS	63.2	50% of therapy	67.3	61.4	6 weeks	69

↓ = significant decrease.

*1 μmol/l = 1 μg/l x 0.0127 [33].

**1 ppm = 1,000 μg/l.

##### Selenium levels

Selenium levels were determined in the plasma, serum or whole blood using atomic absorption spectrometry (AAS) in 9 studies, inductively coupled plasma mass spectrometry (ICP-MS) in 2 studies and a fluorometric method in 1 study. The measurements were performed before radiotherapy, during radiotherapy, at the end of therapy and at specific time points (2 weeks, 6 weeks, 8 weeks or 1 year) after therapy completion.

##### Effect of radiotherapy on selenium levels

The results of selenium levels in patients who underwent radiotherapy without supplementation demonstrated that selenium levels had a tendency to decrease after radiotherapy. In 7 studies (study No. 2, 4, 5, 7, 10, 12, and 15), the selenium levels were not significantly different before and after therapy, but in 3 studies (study No. 1, 8, and 9), the selenium levels after therapy were significantly lower than those before therapy.

#### Effect of selenium supplementation on selenium levels

Table 3 shows the selenium levels in the patients who underwent radiotherapy with selenium supplementation. The selenium levels in patients who received selenium supplementation had a tendency to increase after radiotherapy. However, the selenium levels decreased again at 8 weeks (study No.12) and 6 weeks (study No.15) after the completion of radiotherapy without selenium supplementation.

**Table 3:** Selenium levels in patients who underwent radiotherapy with selenium supplementation

**Study no.**	**Reference**	**Sample**	**Measurement method**	**Mean selenium levels (μg/L)**					
				**Before radiotherapy**	**During radiotherapy**		**End of radiotherapy**	**After completion radiotherapy**	
9	Pakdaman, [27]	Not mentioned	Not mentioned	63	-	-	120 **↑**	-	-
12	Kiremidjian-Schumacher et al. [28]	Plasma	Graphite-furnace AAS	91.29	-	-	8th weeks: 105.29 **↑**	8 weeks (without supplemen-tation)	88.73
15	Muecke et al. [31]	Whole blood	AAS	65.3	50% of therapy	93.2**↑**	90.9 **↑**	6 weeks (without supplemen-tation)	73.2

↑ = increase.

#### Selenium supplementation studies

Table 4 provides a summary of selenium supplementation studies. The selenium supplementation studies in patients who underwent radiotherapy were conducted from 1998 to 2010. Different types of studies were conducted, including a randomized double-blind placebo-controlled study (study No. 12), a multicenter phase 3 trial (study No. 15), and a randomized phase 2 study (study No.16).

**Table 4:** Selenium supplementation studies in patients who underwent radiotherapy

**Study no.**	**Reference**	**Type of cancer/disease**	**Type of study**	**Form of Se used for supplementation**	**Dose (μg)**	**Administration**	**Items observed/measured**	**Result**
11	Pakdaman, [27]	Brain tumor	Not mentioned	Sodium selenite	1000/day (4–8 weeks)	Infusion (during radiotherapy)	Mineral elements, Se and other blood parameters (AST, ALT, γ-GTP, ESR)	A significant diminution of symptoms of intracranial pressure was achieved in 76% of patients.
12	Kiremidjian-Schumacher et al. [28]	Head and neck cancer	Randomized double-blind placebo-controlled study	Sodium selenite	200/day (8 weeks)	Oral (during radiotherapy)	Se in plasma, CTL, MLR, PHA	Significantly enhanced cell-mediated immune responsiveness
13	Micke et al. [29]	Secondary Lymphedema	Not mentioned	Sodium selenite	500/day (4–6 weeks)	Oral (4 or10 months after radiotherapy)	Foldi and Miller scoring and quality of life	Foldi and Miller score: more than 78% showed an improvement of one stage or more
14	Elango et al. [30]	Oral cancer	Not mentioned	Sodium selenite	400/day (6 months)	Oral (during radiotherapy)	Plasma Se, enzymatic (GPx and others) and non-enzymatic antioxidants	Supplementation increased the enzymatic and non enzymatic defense systems
15	Muecke et al. [31]	Cervical cancer (n = 11); Uterin cancer (n = 70)	Multicenter, phase 3 trial	Sodium selenite	500 or 300/day	Oral (during radiotherapy)	Whole blood Se	Statistically significant in reducing the number of episode and severity of RT-induced diarrhea
16	Buntzel et al. [32]	Head and neck (n = 39)	Randomized phase II study	Sodium selenite	500 or 300/day	Oral (during radiotherapy)	Side effect evaluation	Reduced the development of dysphagia due to radiotherapy

##### Therapeutic form of selenium, dose and administration

All of the studies of selenium supplementation in patients who underwent radiotherapy used sodium selenite as the form of selenium for supplementation. Sodium selenite was administered orally in most studies (study No. 12–16) and in physiological saline in another report. The dose of supplementation by oral administration ranged from 200 to 500 μg daily, or 1,000 μg daily by infusion in physiological saline.

##### Parameters observed or measured

To assess the effectiveness of selenium supplementation in radiotherapy, the parameters measured or observed in the studies were selenium levels in the serum, plasma or whole blood; mineral elements in the blood and other blood parameters (aspartate amino transferase (AST), alanine amino transferase (ALT), gamma glutamyl transpeptidase (γ-GTP) and erythrocyte sedimentation rate (ESR)) [27]; immune function [28]; quality of life [29]; enzymatic and non-enzymatic antioxidants [30]; and side effects [31,32].

##### Effects of selenium supplementation on therapy

Most of the studies revealed positive effects of selenium supplementation on the general condition of the patients and their quality of life. The effects of supplementation were different depending on cancer type. No reduction in effectiveness of radiotherapy [31] and no selenium toxicities or complications were reported in any of the supplementation studies.

Pakdaman (study No.11) reported that treatment with sodium selenite in patients with brain tumors was well tolerated by all patients and increased blood selenium levels [27]. A significant diminution of symptoms of intracranial pressure was achieved in 76% of patients after sodium selenite treatment [27].

In the study by Kiremidjian-Schumacher et al. (study No. 12), sodium selenite treatment was shown to significantly enhance cell-mediated immune responsiveness in head and neck cancer patients [28]. This outcome related to the ability of selenium to enhance the expression of both the α- (p55) and β- (p70/75) subunits of the interleukin-2 receptor (IL2-R), which resulted in a greater number of high-affinity IL2-R/cells and enhanced proliferation and differentiation in cytotoxic effector cells [28,34,35].

Micke et al. (study No. 13) demonstrated, using the visual analogue scale, that the self-assessment of the quality of life of patients suffering from head and neck cancer with lymphedema significantly improved after selenium supplementation [29].

Elango et al. (study No. 14) found that supplementation with selenium in oral cancer patients for 6 months may help to increase the enzymatic (superoxide dismutase (SOD), catalase (CAT), glutathione peroxidase (GPx), Glutathione reductase (GRx), Glucose-6-phosphate dehydrogenase (G6PDH)) and non-enzymatic (glutathione (GSH), vitamin E, vitamin C, vitamin A and ceruloplasmin) defense systems. The mechanism of the increase in the activity of the enzymatic defense system is due to increased GPx synthesis as a result of the enhanced *de novo* synthesis of this enzyme in the erythroid precursors of red blood cells [30].

Muecke et al. (study No. 15) reported that selenium supplementation in cervical and uterine cancer patients yielded significant prevention of diarrhea and thus improved the quality of life [31]. The increased activity of the protective intestinal GPx isoenzymes may be responsible for these effects due to the enhanced neutralization of radiation-induced hydroperoxides and free radicals in the small intestinal mucosa included in the radiation volume [31].

In the study by Büntzel et al. (study No.16) selenium supplementation reduced the radiation-associated side-effects of dysphagia developments in patients with head and neck cancer patients [32].

#### Parameters used to assess the effect of radiotherapy on selenium status

We found that the plasma, serum or whole blood selenium levels were common parameters used to assess the effect of radiotherapy on selenium status and the effectiveness of selenium supplementation. Selenium levels had a tendency to decrease after radiotherapy and to increase with selenium supplementation. The mechanism of this decrease is still unclear [24,25]. Pothier et al. suggested that poor dietary intake due to anorexia, nausea, and obstruction, compounded by selenium loss from vomiting, diarrhea, and malabsorption, probably played a role in the decrease [17]. Radiotherapy and chemotherapy, combined with the suboptimal nutrition of cancer patients, may further aggravate the selenium deficiency [24,31]. Muecke et al. highlighted that patients with higher blood selenium levels had a better radiation tolerance, without any effect on the survival data [31]. Therefore, Muecke et al. and Franca et al. strongly recommended that physicians take the selenium status into account before prescribing any anticancer therapy to their patients or consider additional supplementation before therapy when the current selenium status appears insufficient [24,31].

#### Sodium selenite supplementation

In the studies reviewed, sodium selenite was the only form of selenium used for supplementation. In nature, selenium exists in many forms. The most well studied forms are selenomethionine (SeMet), sodium selenite, selenium methylselenocysteine, 1,4,-phenylenebis (methylene) selenocyanate (p-XSC), and methylseleninic acid (MSA) [15]. Selenomethionine and selenocysteine (SeCys) are found predominantly in foods such as bread, cereals, nuts, meat, fish, and other seafood [36]. In human antioxidant systems, selenium participates in the form of SeCys incorporated into the various selenoproteins [11,15]. There are at least 25 known selenoproteins, including GPx, TrxR, iodothyroninedeiodinase, and the selenoproteins P, W and R [37]. The most abundant selenoproteins in the blood are selenoprotein P, which accounts for approximately 50% of plasma selenium [36,38,39], and GPx, which accounts for 10–30% of plasma selenium [36,39].

Sodium selenite, an inorganic form of selenium, was used for supplementation because it can primarily improve the expression of selenoproteins after specific incorporation as SeCys [31]. Sodium selenite also has high biological activity and availability in the body [28] and is known to easily pass the blood–brain barrier [27]. It does not raise the concentrations of nonspecific selenium-containing proteins (e.g., selenium-albumin), which is in contrast to other widely used organic selenium supplements (e.g., selenomethionine) [31].

#### Selenium toxicity

The Food and Nutrition Board, Institute of Medicine, suggested a recommended dietary allowance (RDA) of selenium for both men and women of 55 μg (0.7 μmol)/day [40]. The tolerable upper intake level (UL) of selenium in adults is set at 400 μg (5.1 μmol)/day based on the adverse effect of selenosis [40]. The results of our review of supplementation studies revealed that selenium supplementation doses ranging from 200–500 μg/day by oral administration was well tolerated by all patients, and no toxicities were reported. Selenium supplementation increased the blood selenium level, improved the general condition of patients, improved quality of life and prevented or reduced the side effects of radiotherapy. Muecke et al. [31] also implied that supplementation with selenium neither interferes with the biological effects of ionizing radiation nor protects tumor cells.

The Nutritional Prevention of Cancer (NPC) trial reported that selenium supplementation in subjects, with histories of non-melanoma skin cancers significantly decreased the incidence of lung cancer in patients with low baseline selenium concentrations, but supplementation did not significantly decrease this incidence among individuals in the overall population [41]. Therefore, selenium supplementation may have benefits if the selenium is administered to patients with low selenium levels.

High-dose selenium and long-term supplementation may be ineffective and unsafe because selenium can be toxic at high concentrations. The NPC trial also reported an association between long-term selenium supplementation and an increased risk of diabetes [42]. A review paper in 2006 [43] revealed that serum selenium levels ranging from 400–30,000 μg/l were associated with acute toxicity and that levels ranging from 500–1,400 μg/l were associated with chronic toxicity (mean normal serum selenium level is 125 μg/l [44]).

Another high-dose selenium case consisting of the use of a liquid dietary supplement containing 200 times the labeled concentration of selenium was reported in the United States [44]. Of the 201 cases identified in 10 states, 1 person was hospitalized. The median estimated dose of selenium consumed was 41,749 μg/day. The frequently reported symptoms included diarrhea (78%), fatigue (75%), hair loss (72%), joint pain (70%), nail discoloration (61%) and nausea (58%). The symptoms persisting 90 days or longer included fingernail discoloration and loss (52%), fatigue (35%), and hair loss (29%) [44].

## Conclusion

This paper summarized 16 clinical studies on selenium and radiotherapy conducted from 1987 to 2012. The studies included 1303 cancer patients. To assess the selenium status in patients before and after radiotherapy, the plasma, serum or whole blood selenium level was a common parameter used to assess the effect of radiotherapy on selenium status and the effectiveness of selenium supplementation. Selenium supplementation increased the blood selenium level, improved the general condition of patients, improved quality of life, prevented or reduced the side effects of radiotherapy and did not reduce the effectiveness of radiotherapy or cause any toxicity.

The results of our summary suggest that selenium supplementation in the form of sodium selenite at doses ranging from 200–500 μg daily by oral administration may offer benefits for head and neck cancer; head and neck cancer with lymphedema; and oral, cervical and uterine cancer patients who undergo radiotherapy and have low selenium levels. In the future, further research and additional evidence of the benefits of selenium supplementation in patients during radiotherapy are required to clarify optimal dosing strategies in specific types of cancer and the associated risks, to ensure therapeutic efficacy before it can be recommended for broad clinical use.

## Abbreviations

ROS: Reactive oxygen species; TrxR: Thioredoxin reductases; PKC: Protein kinase C; CAM: Complementary alternative medicine; MeSH: Medical subject headings; AAS: Absorption spectrometry; ICP-MS: Inductively coupled plasma mass spectrometry; AST: Aspartate amino transferase; ALT: Alanine amino transferase; γ-GTP: Gamma glutamyl transpeptidase; ESR: Erythrocyte sedimentation rate; IL2-R: Interleukin-2 receptor; SOD: Superoxide dismutase; CAT: Catalase; GPx: Glutathione peroxidase; GRx: Glutathione reductase; G6PDH: Glucose-6-phosphate dehydrogenase; GSH: Glutathione; SeMet: Selenomethionine; p-XSC: 1,4,-phenylenebis (methylene) selenocyanate; MSA: Methylseleninic acid; SeCys: Selenocysteine; RDA: Recommended dietary allowance; UL: Upper intake level; NPC: Nutritional prevention of cancer.

## Competing interests

The authors have no competing interests to declare.

## Authors’ contributions

IMP, TN and HK were responsible for the study design. IMP, RA, CY and SK were responsible for articles collections and analysis. TN and HK were responsible for supervising the study. All authors participated in the drafting and revising of the manuscript. All authors read and approved the final manuscript.
